# Prenatal phenotype features and genetic etiology of the Williams-Beuren syndrome and literature review

**DOI:** 10.3389/fped.2023.1141665

**Published:** 2023-03-17

**Authors:** Yunan Wang, Chang Liu, Rong Hu, Juan Geng, Jian Lu, Xianzhe Zhao, Ying Xiong, Jing Wu, Aihua Yin

**Affiliations:** ^1^ Medical Genetic Center, Guangdong Women and Children Hospital, Guangzhou, China; ^2^ Maternal and Children Metabolic-Genetic Key Laboratory, Guangdong Women and Children Hospital, Guangzhou, China; ^3^ UItrasonic Diagnosis Deparment, Guangdong Women and Children Hospital, Guangzhou, China

**Keywords:** williams-Beuren syndrome (WBS), prenatal diagnosis, sonographic features, SNP-array, intrauterine phenotypes

## Abstract

**Objective:**

To share our experience on prenatal diagnosis of Williams-Beuren syndrome(WBS) and to improve the awareness, diagnosis, and intrauterine monitoring of the fetuses of this disease.

**Methods:**

The study retrospectively evaluated 14 cases of WBS diagnosed prenatally by single nucleotide polymorphism array (SNP-array). Clinical data from these cases were systematically reviewed, including maternal demographics, indications for invasive prenatal diagnosis, ultrasound findings, SNP-array results, trio-medical exome sequencing (Trio-MES) results, QF-PCR results, pregnancy outcomes and follow-ups.

**Results:**

A total of 14 fetuses were diagnosed with WBS and their prenatal phenotypes were assessed retrospectively. In our case series, the most common ultrasound features were intrauterine growth retardation (IUGR), congenital cardiovascular defects, abnormal fetal placental doppler indices, thickened nuchal translucency(NT) and polyhydramnios. Other less common ultrasound features include fetal hydrops, hydroderma, bilateral pleural effusion, subependymal cysts, etc. Parental chromosome analysis was performed in seven pairs of parents, and all the deletions on chromosome 7q11.23 were *de novo*.

**Conclusion:**

Prenatal ultrasound features of WBS cases are highly variable, with IUGR, cardiovascular abnormalities and abnormal fetal placental doppler indices, being the most common intrauterine phenotypes. Our case series expand the intrauterine phenotypes of WBS, including cardiovascular abnormalities right aortic arch(RAA) combined with persistent right umbilical vein(PRUV) and elevated the ratio of end-systolic peak flow velocity to end-diastonic peak flow velocity(S/D). In the meantime, with the decrease in the cost of the next-generation sequencing, the method may become widely used in prenatal diagnosis in the near future.

## Introduction

Williams–Beuren syndrome (WBS) is a relatively rare microdeletion disease caused by mispairing of low-copy DNA repetitive elements during meiosis. Most patients with WBS have similar deletion sizes resulting in the loss of one copy of 25–27 genes on chromosome 7q11.23 (1), and the typical deletion of the syndrome is 1.4–1.5 Mb. This disorder affects multisystem, and the cardinal features include cardiovascular disease (especially supravalvar aortic stenosis), a specific facial features and a distinctive cognitive and behavioral disability and hyper sociability. But limited information has been collected on the intrauterine phenotype features of WBS. Until now, only about 29 prenatal cases of WBS have been reported (2–11). The prenatal diagnosis of WBS is difficult due to the atypical features of prenatal ultrasound. However, due to the uncertain prognosis of WBS, the detection rate should be improved for timely intrauterine intervention.

In our case series, fourteen fetuses with WBS identified by single nucleotide polymorphism array (SNP-array) are described. We provide clinical features, intrauterine phenotypes and molecular cytogenetic results of the 14 cases, and we compare them with published data to outline distinguishing and shared features. To our knowledge, this study is the most extensive prenatal study of the detailed molecular analysis of WBS cases using chromosomal microarray analysis (CMA) techniques.

## Material and methods

### Subject

We reviewed 14 consecutive fetuses of WBS diagnosed at our Center from January 2016 to September 2021. Clinical data from these cases were systematically reviewed, including maternal demographics, indications for invasive prenatal diagnosis, ultrasound findings, SNP-array results, trio-medical exome sequencing (Trio-MES) results, QF-PCR results, pregnancy outcomes and follow-up data. Each case underwent a routine ultrasound scan at primary hospitals, and referred to our center for reassessment. Their initial indications for invasive prenatal diagnosis include anomalies on ultrasonography (Case 1–12), high risks for large deletion of chromosome 7 (case 13) and high risks for WBS (case 14) by expanded noninvasive prenatal testing (NIPT). After ultrasound reassessment and genetic counseling, all pregnant women received invasive prenatal diagnosis, and the types of prenatal diagnosis were chosen according to their gestational ages (Table 1). Maternal age ranges from 23 to 33 years, with an average of 28.7 years. The gestational age at prenatal diagnosis ranges from 13 to 32 weeks, with an average of 25.6 weeks. Follow-up data were collected on height, weight, facial features, physical activity, and mental responses of newborns. This study has been approved by the Institutional Review Board/ Medical Ethics Committee of Guangdong Women and Children Hospital (IRB reference number: 201801073). Written informed consent was obtained from each participating family.

**Table 1:** Genetic results of all reported fetuses with Williams–Beuren syndrome (WBS).

	NO.	Maternal age	Gestational age at diagnosis	expanded NIPT	Prenatal Diagnosis	Genetic tests	Fetal karyotype	CMA result		Inheritance	Outcome of pregnancy
								CNVs type	Size(Mb)		
*Present cases*	*case 1*	*26*	*31*	*/*	*PUBS*	*SNP array,G-banding,QF-PCR*	*46,XX*	*arr[GRCh37] 7q11.23 (72557179_74628840)x1*	*2*.*1*	*/*	*TOP*
	*case 2*	*29*	*23*	*/*	*Amniocentesis*	*SNP array,G-banding,QF-PCR*	*46,XX*	*arr[GRCh37] 7q11.23 (72624166_74209678)x1*	*1*.*59*	*/*	*TOP*
	*case 3*	*31*	*31*	*low risk*	*PUBS*	*SNP array,G-banding,QF-PCR*	*46,XY*	*arr[GRCh37] 7q11.23 (72701098_74136633)x1*	*1*.*4*	*de novo*	*TOP*
	*case 4*	*29*	*29*	*low risk*	*Amniocentesis*	*SNP array,G-banding,QF-PCR*	*46,XX*	*arr[GRCh37] 7q11.23 (72669480_74154209)x1*	*1*.*5*	*de novo*	*TOP*
	*case 5*	*23*	*32*	*low risk*	*Amniocentesis*	*SNP array,G-banding,Trio-MES*, *QF-PCR*	*46,XY*	*arr[GRCh37] 7q11.23 (72692113_74154209)x1*	*1*.*5*	*de novo*	*Delivery by CS and died a week later*
	*case 6*	*29*	*24*	*low risk*	*PUBS*	*SNP array,G-banding,QF-PCR*	*46,XY,inv(9)(p11q13)*	*arr[GRCh37] 7q11.23 (72723370_74136633)x1*	*1*.*4*	*/*	*TOP*
	*case 7*	*30*	*32*	*/*	*Amniocentesis*	*SNP array,G-banding,QF-PCR*	*46,XY*	*arr[GRCh37] 7q11.23 (72677084_74154209)x1*	*1*.*5*	*/*	*Delivery by CS at 35w3d*
	*case 8*	*26*	*13*	*/*	*CVS*	*SNP array,G-banding,Trio-MES*, *QF-PCR*	*46,XX*	*arr[GRCh37] 7q11.22q21.11 (72081552_77582265)x1*	*5*.*5*	*de novo*	*TOP*
	*case 9*	*27*	*25*	*/*	*Amniocentesis*	*SNP array,G-banding,QF-PCR*	*47,XXX*	*arr[GRCh37] (X) × 3 7q11.23 (72664089_74154209)x1*	*1*.*5*	*/*	*TOP*
	*case 10*	*27*	*29*	*low risk*	*PUBS*	*SNP array,G-banding,Trio-MES*, *QF-PCR*	*46,XY*	*arr[GRCh37] 7q11.23 (72633240_74154209)x1*	*1*.*5*	*de novo*	*TOP*
	*case 11*	*31*	*24*	*/*	*Amniocentesis*	*SNP array,G-banding,Trio-MES*, *QF-PCR*	*46,XY*	*arr[GRCh37] 7q11.23 (72723371_74146927)x1*	*1*.*4*	*de novo*	*TOP*
	*case 12*	*30*	*22*	*high risk for large deletion of chromosome 7*	*Amniocentesis*	*SNP array,G-banding,QF-PCR*	*46,XY,del(7)(q11.2q21)*	*arr[GRCh37] 7q11.21q21.11 (64256482_85672186)x1*	*21*.*4*	*/*	*TOP*
	*case 13*	*31*	*20*	*high risk for WBS*	*Amniocentesis*	*SNP array, G-banding,QF-PCR*	*46,XY*	*arr[GRCh37] 7q11.23 (72718124_74154209)x1*	*1*.*4*	*de novo*	*TOP*
	*case 14*	*33*	*24*	*/*	*Amniocentesis*	*SNP array, G-banding,QF-PCR*	*46,XY,inv(9)(p12q13)*	*arr[GRCh37] 7q11.23 (72636884_74v146927)x1 11q12.1 (56812656_59398190)x3 (VOUS)*	*1*.*5*	*/*	*TOP*
Dadelszen et al., 2000	case 15	25	30	/	/	FISH,G-banding	46,XX, t(6;7)(q27;q11.23)	/	/	/	Died shortly after delivery
Kontos et al., 2008	case 16	34	23	/	Amniocentesis	MLPA,FISH	46,XX	/	/	/	TOP
Krzeminska et al., 2009	case 17	31	20	/	/	FISH	/	/	/	/	TOP
Popowski, Vialard,Leroy, Bault, & Molina, 2011	case 18	22	25	/	/	Prenatal BoBs,FISH	46,XX	/	/	*de novo*	TOP
Marcato et al., 2014	case 19	36	20+2	/		Prenatal BoBs,FISH,array CGH	46,XX	/	/	*de novo*	TOP
	case 20	31	32	/		G-banding,Prenatal BoBs,FISH,array CGH	46,XX	/	/	*de novo*	TOP
	case 21	30	13	/		G-banding,FISH,array CGH	46,XY	/	/	*de novo*	TOP
Kobalka, Mrak, Gunning, 2017	case 22	28	34	/		Not mentioned	/	/	/	/	Stillborn
Srinivasan, Howley, Cuneo,& Chatfield, 2018	case 23	/	34	/		FISH	/	/	/	/	Delivery by CS
MZ Yuan et al. 2019	case 24	35	23	low risk	Amniocentesis	SNP array,G-banding,QF-PCR	46,XY	arr[GRCh37] 7q11.23 (72745047_74138460)x1	1.39	*de novo*	TOP
	case 25	27	22	low risk	Amniocentesis	SNP array,G-banding,QF-PCR	46,XX	arr[GRCh37] 7q11.23 (72732834_74136633)x1	1.4	*de novo*	Delivery by CS
	case 26	37	20	low risk	Amniocentesis	SNP array,G-banding,QF-PCR	46,XY	arr[GRCh37] 7q11.23 (72725759_74154209)x1	1.43	*de novo*	TOP
	case 27	34	23	low risk	Amniocentesis	SNP array,G-banding,QF-PCR	46,XX	arr[GRCh37] q11.23 (72624166_74207565)x1	1.58	*de novo*	TOP
	case 28	33	24+4	low risk	Amniocentesis	SNP array,G-banding,QF-PCR	46,XX	arr[GRCh37] 7q11.23 (72765457_74175640)x1	1.588	*de novo*	DCDA, selective fetocide
	case 29	32	32+2	low risk	Amniocentesis	SNP array,G-banding,QF-PCR	46,XY	arr[GRCh37] 7q11.23 (72621722_74209949)x1	1.504	*de novo*	TOP
	case 30	28	24	low risk	Amniocentesis	SNP array,G-banding,QF-PCR	46,XY	arr[GRCh37] q11.23 (72650120_74154527)x1	1.41	*de novo*	TOP
YH Dang et al. 2019	case 31	16	20	/	Amniocentesis	Prenatal BoBs,SNP array	47, XXY	arr[GRCh37] 7q11.23 (72718123_74154209)x1	1.4	/	TOP
	case 32	31	22	/	Amniocentesis	Prenatal BoBs,SNP array	/	arr[GRCh37] 7q11.23 (72765457_74257046)x1	1.49	*de novo*	TOP
	case 33	24	22	/	Amniocentesis	Prenatal BoBs,SNP array	/	arr[GRCh37] 7q11.23 (72655376_74154209)x1	1.5	*de novo*	TOP
	case 34	31	33+3	/	Amniocentesis	Prenatal BoBs,SNP array	/	arr[GRCh37] 7q11.23 (72713282_74154209)x1	1.44	*de novo*	TOP
	case 35	37	24	/		Prenatal BoBs,SNP array	/	arr[GRCh37] 7q11.23 (72723370_74146927)x1	1.4	*de novo*	TOP
Ruibin Huang et al. 2022	case 36	31	23	/		SNP array,QF-PCR	/	arr[GRCh37] 7q11.23 (72723370_74154209)x1	1.43	/	TOP
	case 37	27	33	/		SNP array,QF-PCR	/	arr[GRCh37] 7q11.23 (72624203_74154497)x1	1.53	/	TOP
	case 38	29	30	/		SNP array,QF-PCR	/	arr[GRCh37] 7q11.23 (72718277_74143060)x1	1.42	/	Lost to follow-up
	case 39	34	22	/		SNP array,QF-PCR	/	arr[GRCh37] 7q11.23 (72718277_74142190)x1	1.42	/	TOP
	case 40	38	33	/		SNP array,QF-PCR	/	arr[GRCh37] 7q11.23 (72718278_74143030)x1	1.42	/	TOP
	case 41	33	31	/		SNP array,QF-PCR	/	arr[GRCh37] 7q11.23 (72557180_74628840)x1	2.07	/	TOP
	case 42	23	27	/		SNP array,QF-PCR	/	arr[GRCh37] 7q11.23 (72701099_74136633)x1	1.44	/	TOP
	case 43	32	24	/		SNP array,QF-PCR	/	arr[GRCh37] 7q11.23 (72723371_74141494)x1	1.42	/	TOP

PUBS, Percutaneous cord blood sampling; CVS, Chorionic Villus Sampling; TOP, termination of pregnancy; CS, cesarean section; DCDA dichorionic diamniotic; VOUS,variants of uncertain significant.

Case 16–23 all accept molecular analysis, including prenatal BoBs analysis or FISH etc., the CNV type were not available, so the molecular results of case 16–23 were not represented in the table.

### Cytogenetic and molecular analyses

G-banding (320–400 bands) was performed on metaphase chromosomes of amniotic fluid cells/ umbilical cord blood lymphocytes/ villus cells using standard procedures (12). Genomic DNA was extracted from fetal uncultured amniotic fluids /umbilical cord blood lymphocytes/ villus cells and their parents' peripheral blood using the Lab-Aid 820 automation system (Zee San Biotech Company, Fujian, China).

SNP-array analysis was performed on a commercial CytoScan 750 K Array (Affymetrix, Santa Clara, CA) containing 750,436 25–85mer oligonucleotide probes, including 550,000 non-polymorphic probes and 200,436 SNP probes (13). The labeling and hybridization of the genomic DNA was performed following the manufacturer's protocol. Results were analyzed by Affymetrix Chromosome Analysis Suite software.

### Medical exome sequencing

Genomic DNA was extracted using a Qiagen DNA blood mini kit (Qiagen GmbH, Hilden, Germany). Library preparation and target enrichment were performed using a SureSelectXT Clinical Research Exome kit (Agilent Technologies, Santa Clara, CA) according to the manufacturer's specifications. Then, Trio-MES was performed using 2 × 150 bp in the paired end mode of the NextSeq 500 platform (Illumina, San Diego, CA) to obtain an average coverage of above 110×, with 97.6% of target bases covered at least 10× (14). Sequence quality analysis and filtering of mapped target sequences were performed with the “varbank” exome and genome analysis pipeline v.2.1 as described previously. Analysis of genetic results was based on the genomic variation database (http://dgv.tcag.ca/dgv/app/home), DECIPHER database (https://decipher.sanger.ac.uk/), and OMIM database (http://www.ncbi.nlm.nih.gov/omim). Found variants were further verified by Sanger sequencing.

## Results

### Intrauterine phenotypes

**Figure 1:**
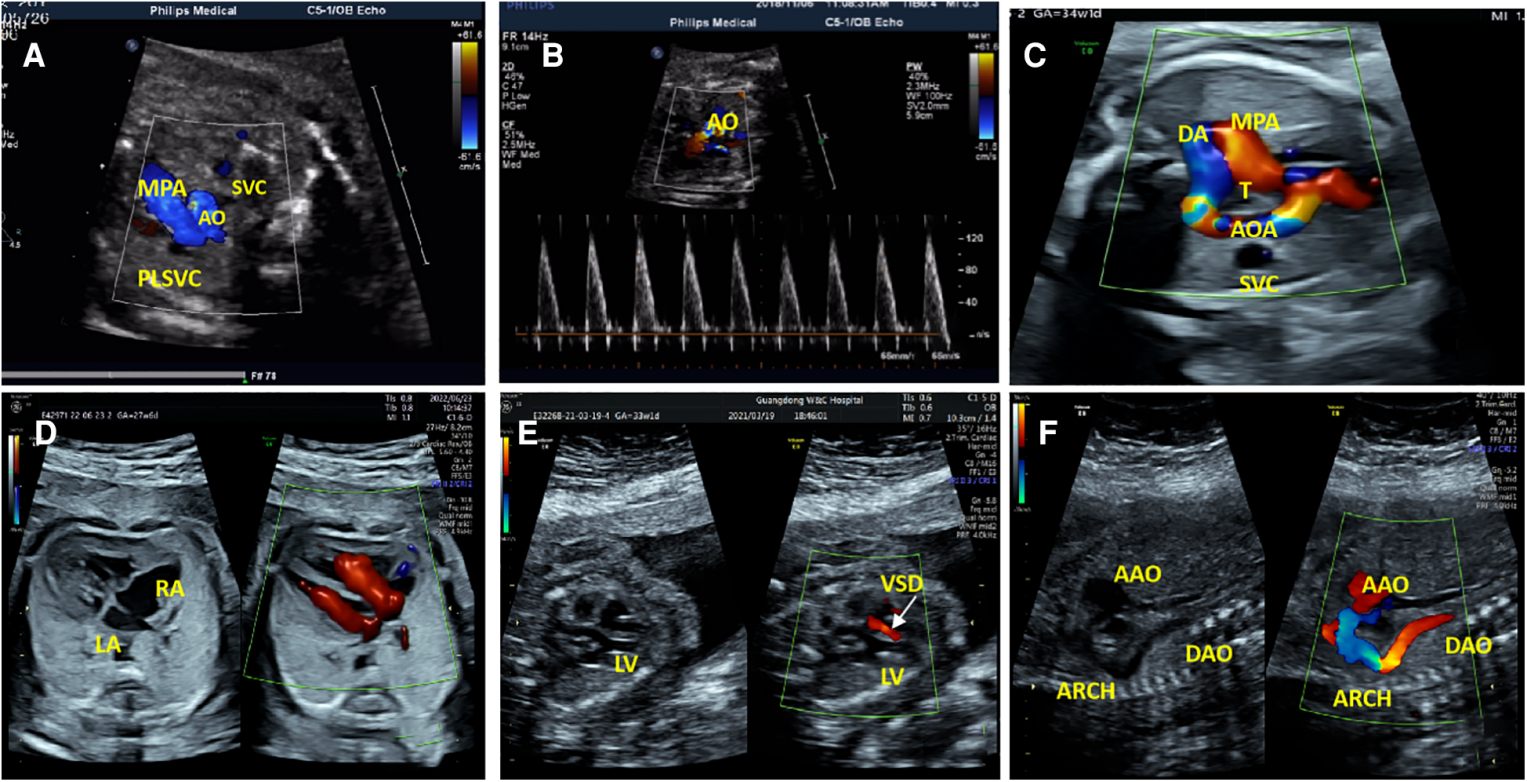
The most common cardiovascular abnormalities in our study. (**A**) The ultrasound examination of case 1 showed a small additional vessel found at the main pulmonary artery (MPA) left in the three-vessel view. SVC, Superior Vena Cava; AO, Aorta; PLSVC, Persistent Left Superior Vena Cava. (**B**)The color Doppler ultrasonic images of case 1 in the left ventricular outflow tract view showed elevated blood flow rate of the aortic valve. (**C**)The ultrasonic examination of case 7 showed that the aortic arch was on the right side of the trachea (**T**), the MPA was on the left side of the T, and they constituted U-shaped ring of blood vessels at the three-vessel view. AOA, Aortic Arch; DA, Ductus Arteriosus. (**D**) Two-dimensional and color Doppler ultrasonic images of case 9 showed enlargement of the right atrium (RA) in the four-chamber view. (**E**)Two-dimensional and color Doppler ultrasonic images of case 14 showed left ventricular septal defect (VSD), and left-to-right shunt of defect part (color Doppler flow signal at the defect) in the left ventricular outflow tract view. LV, Left Ventricle. (**F**) Two-dimensional and color Doppler ultrasonic images of case 14 showed that the descending aortic arch extends to the left obviously in the long axis of the aortic arch view. AAO, Ascending Aorta; DAO, Descending Aorta.

**Figure 2:**
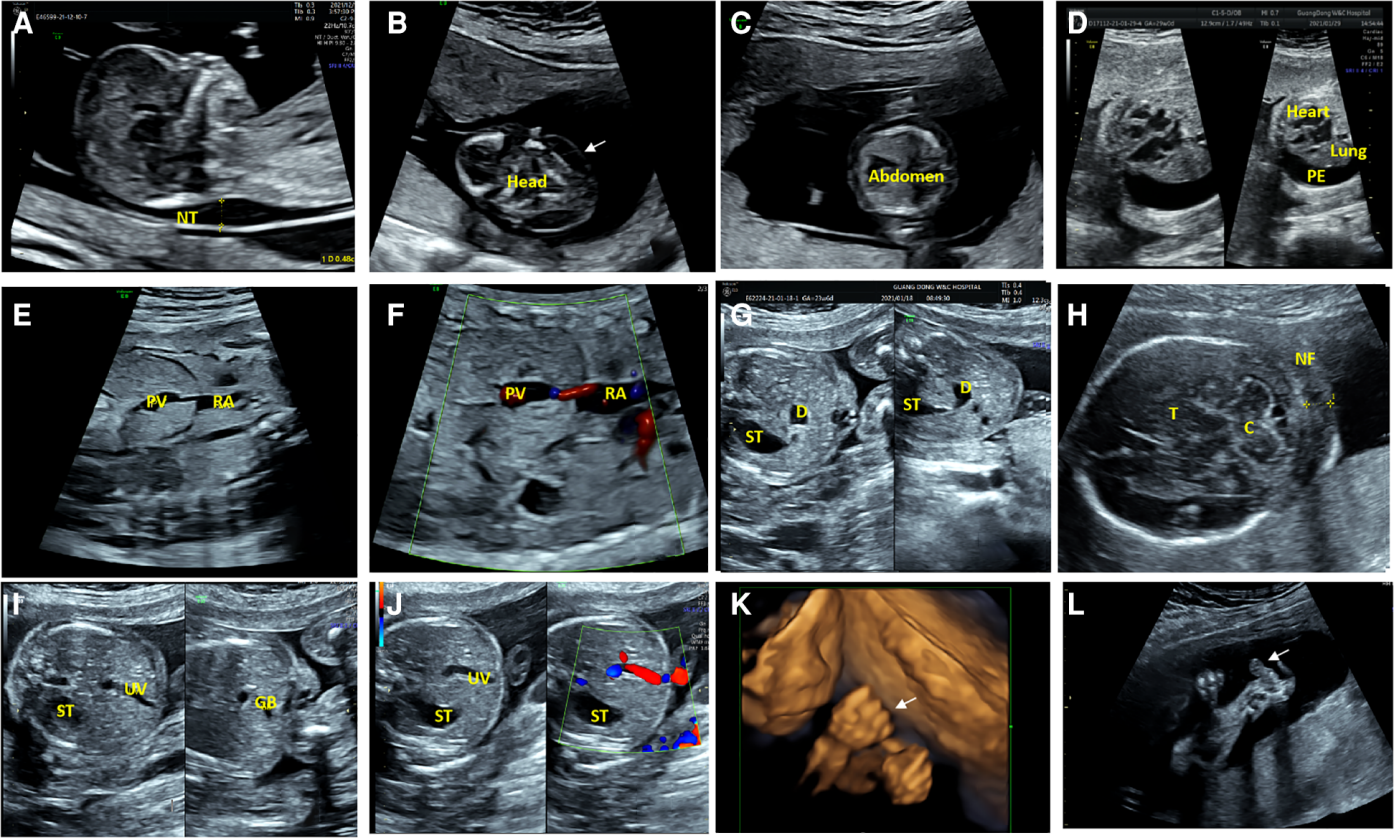
Less common ultrasound features in our study. (**A**) The ultrasonic examination of case 8 showed a thickened nuchal translucency (NT) in the midsagittal view. (**B,C**) The transverse view of the fetal brain and abdomen of case 8 showed hydroderma (the white arrow). (**D**) The ultrasound examination of case 10 showed a left pleural effusion (PE), and dextroposition of heart in the four-chamber view. (**E,F**) Two-dimensional and color Doppler ultrasonic images of case 9 showed the absence of venous duct and abnormal connection between the portal vein (PV) sinus and the right atrium (RA) in the oblique coronal view. (**G**)The transverse view of the upper abdomen of the fetus 11 presented a typical double bubble sign. The anechoic region on the left side of the abdomen is the stomach (ST) and the anechoic region on the right is the expanded duodenum (**D**). (**H**) The ultrasonic examination of the case 12 showed a thickened nuchal fold (NF) in the transverse view of cerebellar. C, Cerebellum; T, Thalamus. (**I,J**) Two-dimensional and color Doppler ultrasonic images of the fetus 11 showed that the umbilical vein(UV) runs towards to the stomach(ST), and the gallbladder(GB) is located to the left of the umbilical vein in the transverse view of the abdomen. (**K**) Three-dimensional ultrasonic images of the fingers showed the hands of case 12 presented in a continuous “Thumb-in-Fist Posture”. (**L**) The ultrasonic examination of case 12 showed that thumbs were continuously adducted, and hypoechoic mass presented in the left radial volar.

Fourteen fetuses were diagnosed with WBS. Their prenatal phenotype features were reviewed retrospectively. Clinical characteristics and genetic results are shown in Table 1. In our case series, the most common ultrasound features were as follows: intrauterine growth retardation (IUGR) (9/14, 64.3%), congenital cardiovascular defects (6/14, 42.9%), abnormal fetal placental doppler indices (7/14, 50%), thickened NT (2/14, 14.3%) and polyhydramnios (2/14, 14.3%). The most common cardiovascular abnormalities in our study encompass vascular ring (3/6, 50%), ventricular septal defect (VSD) (1/6, 16.7%), persistent left superior vena cava (PLSVC)(1/6, 16.7%), enlarged right atrium (1/6, 16.7%), abnormal connection between the portal vein (PV) sinus and the right atrium (RA) (1/6, 16.7%), elevated blood flow rate of the aortic valve (1/6, 16.7%), abnormal extending of descending aortic arch (extends to the left obviously) (1/6, 16.7%). Figure 1 shows some typical ultrasound features of cardiovascular abnormalities in these cases. Abnormal fetal placental doppler indices in our study include elevated ratio of end-systolic peak flow velocity to end-diastonic peak flow velocity of umbilical artery (S/D) (5/7, 71.4%), absence of diastolic of middle cerebral artery (MCA) (1/7, 14.3%) and absence of diastolic of umbilical artery (UA) (1/7, 14.3%). Other less common ultrasound features include persistent right umbilical vein (PRUV) (1/14, 7.1%), fetal hydrops (1/14, 7.1%), hydroderma (1/14, 7.1%), left pleural effusion(1/14, 7.1%), bilateral subependymal cysts(1/14, 7.1%), thickened nuchal fold (NF) (1/14, 7.1%), absence of venous duct (1/14, 7.1%), duodenal atresia (DA) (1/14, 7.1%), low-lying conus medullaris (1/14, 7.1%), and abnormal posture of hands (1/14, 7.1%) (Table 2). Figure 2 shows some less common ultrasound features in our study.

**Table 2:** Intrauterine phenotype features of all reported fetuses with williams–beuren syndrome (WBS).

	NO.	Intrauterine abnomalities																	
		IUGR	Congenital cardiovascular defects	Abnormal fetal placental Doppler indices	Thickened NT /NF	Polyhydramnios	Bilateral subependymal cysts	fetal hydrops/hydroderma/pleural effusion	Absence of venous duct	DA	low-lying CM	Limb abnormalities	Echogenic bowel	IEF	SUA	Omphalocele	Nasal bone dysplasia	Fetal right nasolacrimal duct cyst	Renal cyst
Present cases	case 1	+	PLSVC, elevated blood flow rate of the aortic valve	−	−	−	−	−	−	−	−	−	−	−	−	−	−	−	−
	case 2	+	−	elevated S/D,absence of diastolic of MCA	−	−	−	−	−	−	−	−	−	−	−	−	−	−	−
	case 3	+	−	elevated S/D	Thickened NT	−	−	−	−	−	−	−	−	−	−	−	−	−	−
	case 4	+	−	−	−	−	−	−	−	−	−	−	−	−	−	−	−	−	−
	case 5	+	−	elevated S/D	−	−	−	−	−	−	−	−	−	−	−	−	−	−	−
	case 6	−	−	elevated S/D	−	+	+	fetal hydrops	−	−	−	−	−	−	−	−	−	−	−
	case 7	+	Vascular ring:RAA	−	−	−	−	−	−	−	−	−	−	−	−	−	−	−	−
	case 8	−	Vascular ring	−	Thickened NT	−	−	hydroderma	−	−	−	−	−	−	−	−	−	−	−
	case 9	+	enlarged right atrium	Absence of diastolic of UA	−	−	−	−	+	−	−	−	−	−	−	−	−	−	−
	case 10	−	−	−	−	+	−	Left pleural effusion	−	−	−	−	−	−	−	−	−	−	−
	case 11	+	Vascular ring:RAA;PRUV	elevated S/D	−	−	−	−	−	+	−	−	−	−	−	−	−	−	−
	case 12	−	−	−	Thickened NF	−	−	−	−	−	+	+	−	−	−	−	−	−	−
	case 13	−	−	−	−	−	−	−	−	−	−	−	−	−	−	−	−	−	−
	case 14	+	VSD	−	−	−	−	−	−	−	−	−	−	−	−	−	−	−	−
Dadelszen et al., 2000	case 15	−	Severe SVAS,PAS	Absence of diastolic of UA	−	+	−	fetal hydrops	−	−	−	−	−	−	−	−	−	−	−
Kontos et al., 2008	case 16	−	small VSD	−	−	−	−	−	−	−	−	−	−	−	−	−	−	−	−
Krzeminska et al., 2009	case 17	+	SVAS	−	−	−	−	−	−	−	−	−	+	−	−	−	−	−	−
Popowski, Vialard,Leroy, Bault, & Molina, 2011	case 18	+	small SVAS	−	−	−	−	−	−	−	−	−	−	−	−	−	+	−	−
Marcato et al., 2014	case 19	+	−	−	−	−	−	−	−	−	−	−	+	−	−	−	−	−	−
	case 20	+	−	−	−	−	−	−	−	−	−	−	−	−	−	−	−	−	−
	case 21	+	−	−	−	−	−	−	−	−	−	−	−	−	−	+	−	−	−
Kobalka, Mrak, Gunning, 2017	case 22	−	AC,cardiomegaly	−	−	−	−	−	−	−	−	−	−	−	−	−	−	−	−
Srinivasan, Howley, Cuneo,& Chatfield, 2018	case 23	+	SVAS,bilateral PAS	−	−	−	−	−	−	−	−	−	−	−	−	−	−	−	−
MZ Yuan et al. 2019	case 24	+	VSD	−	−	−	−	−	−	−	−	−	−	−	−	−	−	−	−
	case 25	+	−	−	−	−	−	−	−	−	−	−	−	+	+	−	−	−	−
	case 26	+	−	−	−	−	−	−	−	−	−	−	−	−	−	−	−	−	−
	case 27	+	VSD	−	−	−	−	−	−	−	−	−	−	−	−	−	−	−	−
	case 28	+	AC,PLSVC	−	−	−	−	−	−	−	−	−	−	−	−	−	−	−	−
	case 29	+	−	−	−	−	−	−	−	−	−	−	−	−	−	−	−	−	−
	case 30	+	RAA	−	−	−	−	−	−	−	−	−	−	−	−	−	−	−	−
YH Dang et al. 2019	case 31	−	−	−	−	−	−	−	−	−	−	−	−	−	−	−	−	+	−
	case 32	−	−	−	Thickened NF	−	−	−	−	−	−	−	−	−	−	−	−	−	−
	case 33	−	Coronary veins widened, small amount of pericardial effusion	−	−	−	−	−	−	−	−	−	−	+	−	−	−	−	−
	case 34	−	Fetal right ventricle slant, tricuspid regurgitation	−	−	−	−	−	−	−	−	−	−	−	−	−	−	−	−
	case 35	−	−	−	−	−	−	−	−	−	−	−	−	−	−	−	−	−	+
Ruibin Huang et al. 2022	case 36	−	−	−	−	−	−	−	−	−	−	−	−	−	−	−	−	−	+
	case 37	−	AC	−	−	−	−	−	−	−	−	−	−	−	−	−	−	−	−
	case 38	−	−	−	−	−	−	−	−	+	−	−	−	−	−	−	−	−	−
	case 39	−	TOF, SVAS, RAA	−	−	−	−	−	−	−	−	−	−	−	−	−	−	−	−
	case 40	−	VSD, AC	−	−	−	−	−	−	−	−	−	−	−	−	−	−	−	−
	case 41	+	PAS	−	−	−	−	−	−	−	−	−	−	−	−	−	−	−	−
	case 42	+	−	−	−	−	−	−	−	−	−	−	−	−	−	−	−	−	−
	case 43	−	VSD	−	−	−	−	−	−	−	−	−	−	−	−	−	−	−	−

IUGR, intrauterine growth retardation; VSD, ventricular septal defect; AC, aortic coarctation; PLSVC, persistent left superior vena cava; RAA, right aortic arch; SVAS, supravalvular aortic stenosis; PAS, pulmonary artery stenosis; SUA, single umbilical artery; IEF, intracardiac echogenic focus; +, feature present; −, feature absent.

### Cytogenetic and molecular analyses

**Figure 3:**
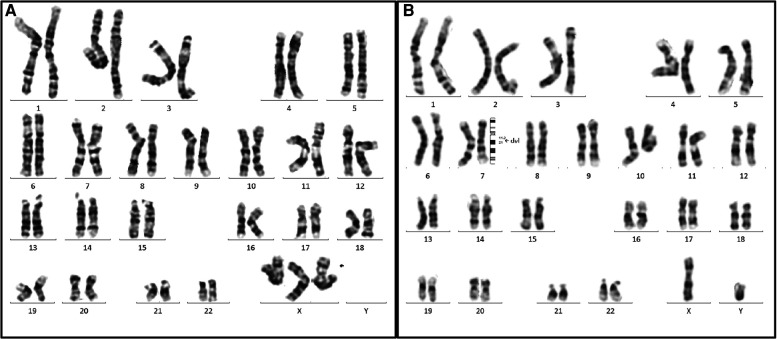
Two abnormal karyotypes in our case series. (**A**) The karyotype on the left is for the case 9 (47, XXX), the arrow indicated the extra X chromosome. (**B**) The karyotype on the right is for case 12. The arrow elicited that the breakpoints of the deletion were located between 7q11.2 and 7q21, and the karyotype was described as 46,XY, del(7)(q11.2q21).

**Figure 4:**
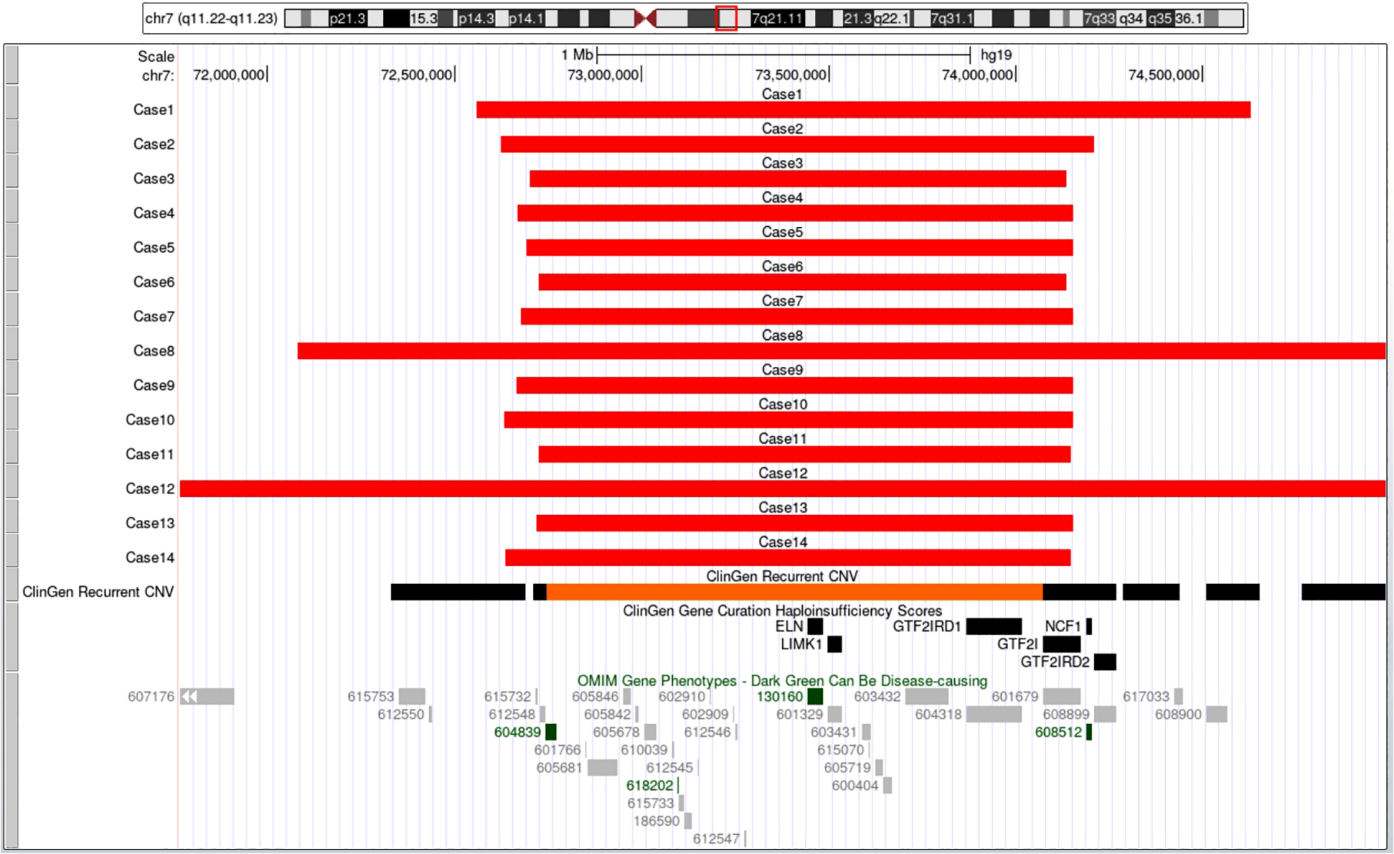
The breakpoints and covered genes of each microdeletion of the 14 fetuses, including the well-known OMIM disease-causing gene *ELN* (130,160) and NCF1 (608,512). Eleven cases had common deletion sizes ranged from 1.4 to 1.5 Mb. Case 1 had 2.1Mb deletions at 7q11.22-q21.11, involved 40 protein-coding genes including *ELN* (130,160). Case 8 had 5.5Mb deletions at 7q11.22-q21.11, involved 71 protein-coding genes including *ELN* (130,160). Case 12 had 21.4Mb deletions at 7q11.21-q21.11, involving 93 protein-coding genes, among which 82 genes including *AUTS2* (607,270) and *ELN* (130,160) were OMIM genes.

All 14 fetuses underwent G-banded karyotype analysis, two abnormal karyotypes were found: 47, XXX (Case 9), 46, XY,del(7)(q11.2q21) (Case 12) (Figure 3). Besides of these, SNP-array analysis found deletions encompassing the WBS critical region (WBSCR) in all 14 fetuses. They had different sizes and loci of chromosome microdeletion, eleven cases had common deletion sizes, ranging from 1.4 to 1.5 Mb. Case 1 had a 2.1 Mb deletion at 7q11.22-q21.11, involved 40 protein-coding genes. Case 8 had a 5.5 Mb deletions at 7q11.22-q21.11, involved 71 protein-coding genes. Case 12 had a 21.4 Mb deletion at 7q11.21-q21.11, involving 93 protein-coding genes. The deletions of the 14 cases are shown in Figure 4.

Case 3, 5, 8, 10 and 11 received Trio-MES additionally, but no more pathogenic variant was found. Another two pairs of parents (Case 4 and 13) accepted CMA analysis. These results indicated that all deletions on chromosome 7q11.23 in the seven cases (Case 3, 4, 5, 8, 10, 11 and13) were *de novo*.

### Follow ups

After detailed genetic counseling, twelve families (Case 1–4, 6, 8–14) decided to terminate the pregnancies. All aborted fetuses had typical WBS facial features, including low nasal bridge, palpebral edema, long philtrum, wide mouth, big ears, small chin, and retrognathia.

Case 5 was delivered at 33^+3^ gestational age by cesarean section (CS) for fetal distress, and his birth weight was 1.32 kg. The newborn had distinctive facial features, including wide forehead, periorbital fullness, epicanthal folds, flat nasal bridge, a short upturned nose, long philtrum, and wide mouth. The newborn was transferred to the Neonatal Intensive Care Unit (NICU) for very low birth weight and respiratory distress, and died a week later for severe respiratory distress syndrome. Case 7 was delivered at 35^+3^ gestational age by CS for fetal distress, and his birth weight was 1.7 kg. The APGAR scores were 9, 10, and 10 at 1, 5, and 10 min, respectively. His appearances are consistent with representative facial phenotypes including straight and neat eyebrows, high wide nose, and broad forehead. He was transferred to the NICU for very low birth weight and respiratory distress, and was discharged after 27 days of symptomatic treatment. Regular check-ups were made. His height and weight were below 3 percentile at two years old. His cardiac evaluation showed RAA, but no other cardiac symptoms for the time being.

## Discussion

Williams–Beuren syndrome (WBS) is a relatively rare microdeletion disorder, manifests as cardiovascular disease, intellectual disability, behavioral and cognitive abnormalities, developmental delay, renal anomalies, and characteristic facial features. Cardiovascular abnormalities, such as suvalvular aortic stenosis (SVAS) and stenosis of other large arteries, are the main cause of morbidity and mortality, and occur in 50%–80% of patients with WBS (15). Prenatal ultrasound diagnosis is relatively difficult for WBS. Various cardiovascular anomalies, ranging from ventricular septal defect to elastin arteriopathy (for example, SVAS), can theoretically be detected by prenatal ultrasound but in clinical practice it is quite difficult (9). Another common prenatal finding of WBS is IUGR, but IUGR is nonspecific and can result from a variety of maternal, fetal, and placental conditions. As a consequence, prenatal ultrasound features of WBS remain incomplete and atypical.

Since the first prenatal WBS case reported in 2009, a total of twenty-nine prenatal cases have been described previously. Here we report 14 cases of WBS diagnosed prenatally by SNP array. Prenatal ultrasound findings of WBS are diversified, ranging from almost no manifestations (Case 13 in our study, at 20 weeks of gestation) to multiple malformations (Case 11 and 12 in our study). Although it is difficult to make a clear diagnosis of WBS in prenatal ultrasound, we may be able to summarize some specific prenatal phenotypes from these 43 cases to provide some insights for the prenatal diagnosis of WBS. The fetuses with WBS manifest IUGR (55.8%, 24/43), abnormal fetal placental Doppler indices(16.3%, 7/43), cardiovascular abnormalities (53.5%, 23/43), which encompass SVAS (21.7%, 5/23), VSD (26.1%, 6/23), AC (17.4%, 4/23), vascular ring(21.7%, 5/23), PAS(8.7%, 2/23), PLSVC(8.7%, 2/23), and TOF (4.3%, 1/23). Some other rare ultrasound findings include thickened NT/ NF(9.3%, 4/43),, polyhydramnios (7.0%, 3/43)fetal hydrops (4.7%, 2/43), duodenal atresia (4.7%, 2/43), echogenic bowel (4.7%, 2/43), intracardiac echogenic focus (IEF) (4.7%, 2/43), renal cyst (4.7%, 2/43), absence of venous duct (2.3%, 1/43), omphalocele (2.3%, 1/43), nasal bone dysplasia (2.3%, 1/43), single umbilicalartery (SUA) (2.3%, 1/43), bilateral subependymal cysts (2.3%, 1/43). Most intrauterine features described in the present case series are consistent with the reports in literature.

Cardiovascular abnormalities, RAA combined with PRUV and elevated S/D, were first described in our study. IUGR and cardiovascular abnormalities are the most common prenatal features of WBS. SVAS and VSD are the most common cardiac defects, followed by vascular lesion of vascular ring. The vascular ring could be diagnosed as early as 13 weeks in our study. Moreover, we found seven WBS fetus with the intrauterine features of abnormal fetal placental doppler indices, including five with elevated S/D, one with absence of diastolic of MCA and one with absence of diastolic of umbilical UA. The S/D, RI and PI values of the UA and MCA could reflect the resistance of blood vessels during blood circulation (16). When the S/D, PI, RI values of the MCA blood flow decreased gradually, and the UA resistance index shows a gradual upward trend, especially when the end-diastolic blood flow loss waveform appeared, it could predict the occurrence of fetal distress (17). Elastin is the major component of the extracellular matrix in many tissues. In terms of vascular development, it plays a role in arterial wall development by regulating smooth muscle proliferation. Elastin deficiency induces excessive proliferation of these cells, leading to arterial wall remodeling and obstructive vascular disease (18). We speculate that the haplo-insufficiency of *ELN* may be relevant to the hemodynamic changes of fetuses with WBS. Summarizing the prenatal phenotypes of WBS would provide insights for ultrasound detection, and may lead to early diagnosis and active treatment for patients at risk.

Currently, the most widely used techniques to detect microdeletion include FISH, multiplex ligation-dependent probe amplification (MLPA) and chromosomal microarray analysis (CMA). FISH has the disadvantage of labor-intensive and time-consuming, the exact size of detected CNVs cannot be determined, and cannot be used in atypical CNVs cases. MLPA assay has been utilized to screen for unknown CNVs, but MLPA also has disadvantages: the relatively small number of probes cover only a few exons and regulatory regions in WBSCR, and since non-overlapping and widely scattered probes are used, breakpoints often cannot be defined precisely. Within the last 10 years, fetal genetics has advanced further with the widespread use of CMA. In addition to provide precise breakpoints for deletion boundaries and provide detection of atypical deletions, CMA can also determine additional CNV elsewhere in the genome. Furthermore, next-generation sequencing may eventually be utilized to perform combined single-nucleotide polymorphism, CNV and structural variant detection in fetal samples. Trio-MES also contains the parental detection, which may assist in determining parental inheritance. With the decrease of the cost, next-generation sequencing may become widely used in prenatal diagnosis.

## Conclusions

In summary, prenatal ultrasound findings of WBS cases are diversified, with IUGR, cardiovascular abnormalities and abnormal fetal placental doppler indices being the most common intrauterine phenotypes. Our case series may help expand the prenatal phenotypes of WBS, including cardiovascular abnormalities RAA combined with PRUV and elevated S/D. These findings may provide insights for possible prenatal diagnosis of WBS by high-resolution ultrasound.In the meantime, with the decrease of the cost, next-generation sequencing may become widely used in prenatal diagnosis.

## Data Availability

The original contributions presented in the study are included in the article/Supplementary Materials, further inquiries can be directed to the corresponding author.
